# Prevalence and Significance of Incidental Findings in Multiparametric Magnetic Resonance Imaging of the Prostate

**DOI:** 10.3390/tomography11110118

**Published:** 2025-10-23

**Authors:** David Weiß, Arne Bischoff, Michael Brönnimann, Matteo Haupt, Martin Maurer

**Affiliations:** 1 University Institute for Diagnostic and Interventional Radiology, Carl von Ossietzky Universität Oldenburg, 26129 Oldenburg, Germany; 2 Department of Diagnostic, Interventional and Pediatric Radiology, Inselspital, Bern University Hospital, University of Bern, 3010 Bern, Switzerland; michael.broennimann@insel.ch

**Keywords:** prostate, incidental findings, radiology, multiparametric MRI

## Abstract

**Objective:** This study aims to assess the prevalence of clinically significant incidental findings as well as incidental findings of minor clinical significance in multiparametric MRI (mpMRI) of the prostate. **Materials and Methods:** A retrospective analysis was conducted on 607 male patients (mean age: 72 years) who underwent prostate MRI between 2018 and 2023 at a single center. Two radiologists reviewed in consensus the scans for incidental findings during multiparametric MRI of the prostate. The findings were classified according to their clinical relevance, organ group and patient age. **Results:** Among 607 male patients (mean age: 72 years), 665 incidental findings were identified in 410 patients (67.5%; 95% CI 63.7–71.1). This corresponds to an average of 1.10 incidental findings per patient across the entire cohort. Of the 665 findings, 12 (1.8%; 95% CI 0.9–3.1) were classified as clinically significant. These included cases of sarcoma, rectal carcinoma, hydronephrosis, aortic aneurysm, avascular necrosis of the femoral head and high-grade disc protrusion with spinal canal stenosis and diverticulitis. **Conclusions:** Our data indicate that incidental findings are common in prostate mpMRI examinations; however, only a small proportion are clinically significant. This underscores the need for awareness of such findings, while avoiding unnecessary follow-up for those without clinical relevance.

## 1. Introduction

Prostate cancer is a major health concern for men worldwide. According to the World Cancer Research Fund, an estimated 1,467,854 new cases were diagnosed globally in 2022, making it the second most common cancer among men [1]. In Germany alone, approximately 65,820 new cases of prostate cancer were diagnosed in 2020 [2]. It remains a leading cause of cancer-related mortality among men, with 15,403 deaths reported in Germany in 2021. Prognosis for prostate cancer varies considerably depending on the stage at diagnosis: while patients with localized disease have a five-year survival rate nearing 100%, those with metastatic disease face a significantly lower five-year survival of approximately 30% [3,4].

Magnetic resonance imaging (MRI) of the prostate—particularly multiparametric MRI (mpMRI)—has become a cornerstone of prostate cancer diagnostics due to its high sensitivity and specificity for detecting clinically significant lesions [5,6]. As the availability of MRI increases and the population ages, the number of mpMRI scans performed continues to rise [7], leading to a greater frequency of incidental findings (IFs) detected outside the prostate. These IFs may vary widely in clinical relevance, ranging from benign or indeterminate lesions to potentially serious pathologies requiring further workup.

Although many IFs are benign, their detection can lead to unintended consequences, including patient anxiety, unnecessary diagnostic procedures, increased healthcare costs, and even iatrogenic harm—particularly when the findings are not clinically significant. This raises important concerns about the potential public health impact of overdiagnosis and overtreatment associated with IFs. Despite these concerns, relatively few studies have systematically evaluated the prevalence and clinical significance of incidental findings in mpMRI performed for prostate cancer assessment.

In parallel, advances in computer-aided detection and artificial intelligence have been applied to prostate mpMRI, ranging from classical machine-learning–based CAD systems to more recent deep-learning approaches. Recent work has reviewed multimodality imaging applications and proposed advanced CAD systems using diffusion-weighted imaging to improve prostate cancer detection [8,9,10]. While these approaches focus on prostatic lesion detection and grading rather than incidental findings, they underscore the broader technological momentum surrounding prostate MRI.

Previous research, such as by Sherrer et al. and Cutaia et al. [11,12], has highlighted the prevalence and range of IFs on prostate MRI, with varying rates of clinical significance. However, more data are needed from diverse clinical settings to better understand their implications and guide clinical decision-making.

Importantly, incidental findings in prostate mpMRI represent a diagnostic challenge due to their frequently unexpected nature and heterogeneous characteristics. Some findings may reflect early-stage malignancies or clinically significant conditions requiring prompt intervention, while others might be benign or clinically insignificant. This variability necessitates a clear framework for assessing and managing these findings to avoid unnecessary patient burden and healthcare expenditure. Furthermore, demographic factors such as patient age, comorbidities, and clinical history may influence the likelihood and significance of incidental findings, underscoring the need for stratified analyses in this context.

Given the expanding use of mpMRI in prostate cancer diagnostics, understanding the epidemiology of incidental findings—especially in relation to their clinical relevance—is essential for optimizing patient management. Our study therefore aims to contribute to this growing evidence base by reporting findings from a consecutive single-center cohort over a five-year period, with a focus on the prevalence, anatomical distribution, and clinical significance of incidental findings. By providing detailed stratification by organ system and age, our work complements prior reports and adds granularity to the current understanding of incidental findings in prostate mpMRI.

## 2. Materials and Methods

The study cohort initially included 648 multiparametric MRI (mpMRI) examinations of the prostate performed between 2018 and 2023, comprising 420 in domo and 228 ex domo scans. Eligible patients were adult males (≥18 years) who underwent pre-biopsy mpMRI at our institution or were referred with external mpMRI examinations for targeted biopsy due to suspected prostate malignancy.

Only examinations that included all essential mpMRI sequences—T2-weighted (T2W), diffusion-weighted imaging/apparent diffusion coefficient (DWI/ADC), and dynamic contrast-enhanced (DCE)—and met a minimum diagnostic quality threshold (PI-Qual v2 ≥ 2) [13] were retained. Examinations were excluded if performed after biopsy, if they represented duplicate scans from the same patient, in which case only the first was included, native without contrast or if sequences were incomplete or technically non-diagnostic.

Initial MRI interpretation followed the Prostate Imaging Reporting and Data System (PI-RADS) version 2.1 from 2019 onward, and version 2.0 for earlier examinations [14,15].

A total of 41 scans were excluded: 13 in domo scans due to the absence of contrast, and 28 ex domo scans primarily because they were incomplete or were performed without contrast.

**Figure 1:**
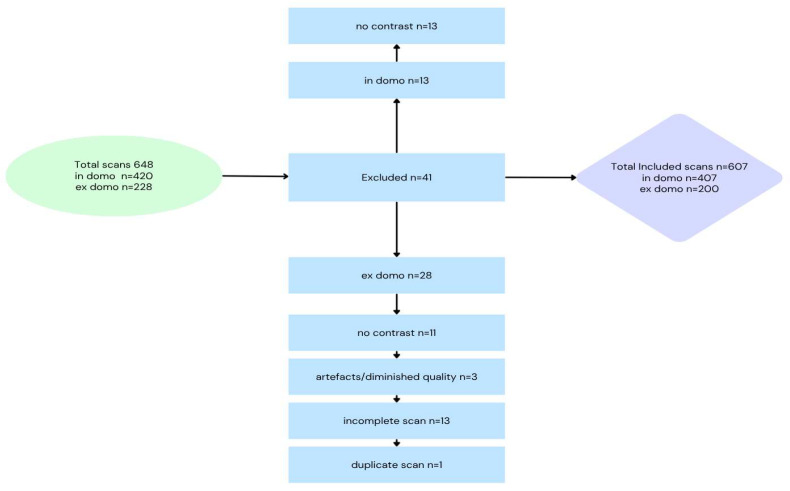
Flowchart Depicting Study Cohort Derivation and MRI Exclusion Criteria.

Altogether, mpMRI examinations from 607 patients were included (Figure 1), consisting of two groups: (1) in-house patients (n = 407), referred based on clinical suspicion of prostate cancer due to abnormal findings such as suspicious digital rectal examination (DRE), transrectal ultrasound, or elevated prostate-specific antigen (PSA) levels [16]; and (2) external mpMRI examinations (n = 200) from patients referred to our institution for further diagnostic workup, including targeted biopsy, following detection of suspicious lesions on prior imaging.

In-house MRI scans were performed using a 1.5 Tesla MRI scanner (Siemens Aera, Siemens Healthineers, Erlangen, Germany), reflecting the available equipment at our institution and ensuring consistency across the study period. To improve image quality and reduce motion artifacts, intravenous Buscopan^®^ (Butylscopolamin; PANPHARMA GmbH (Trittau, Germany)) was routinely administered prior to each in domo scan unless contraindications were present [17]. No adverse effects or worsening urinary dysfunction were observed after the scan in our institution. Our imaging protocol included following sequences: T2-weighted (T2W) HASTE sequences in the transverse, coronal, and sagittal planes; diffusion-weighted imaging (DWI) with apparent diffusion coefficient (ADC) mapping in the transverse plane; T1-weighted (T1W) sequences in the transverse plane; and T1-weighted fat-saturated post-contrast sequences in the transverse plane following intravenous administration of Gadovist^®^ (gadobutrol; Bayer Vital GmbH, Leverkusen, Germany), including dynamic contrast-enhanced (DCE) T1-weighted sequences [15].

Two experienced radiologists (18 and 4 years of work experience in clinical radiology) independently re-read all internal and external MRI scans specifically for this study, blinded to biopsy results and with a predefined focus on extra-prostatic incidental findings (IFs). Discrepancies were resolved in consensus.

IFs were defined as abnormalities unrelated to prostate cancer, excluding findings such as seminal vesicle invasion, rectal invasion, bladder invasion, bone metastases, or pathological lymph nodes, which are typically associated with the primary disease process.

Incidental findings were further categorized according to their clinical relevance and grouped by organ system. Clinically significant findings were defined as an unexpected abnormality identified outside the prostate gland during imaging which has potential clinical relevance and may require further evaluation or management. These findings are not related to prostate malignancy or metastatic disease, but instead involve adjacent pelvic structures that could impact patient care. Examples include bladder wall thickening suggestive of pathology, rectal or colonic abnormalities, influencing diagnosis which may prompt additional investigations and initiate or alter treatment plans.

Non- significant findings were those that did not require any further medical intervention or monitoring. This classification was critical to distinguish incidental findings that could impact patient management from those that were unlikely to affect clinical outcomes.

Due to the relatively small sample sizes and uneven distribution of patients in the younger age brackets, individuals aged 55 years and below were consolidated into a single (Table 1), broader category for the purposes of statistical analysis. This decision was justified, as the limited representation of younger patients would have otherwise compromised the reliability and validity of age-based subgroup comparisons. By combining these younger age groups, we aimed to mitigate the risk of skewed or unstable results. This approach aimed for a more robust assessment of potential age-related trends in the prevalence and clinical relevance of incidental findings, despite demographic imbalances in the dataset.

Interestingly, the analysis revealed a disproportionately high frequency of clinically significant incidental findings in both the 30–55 and >85-year age groups. However, these results are likely attributable to the very small number of patients in these categories—only 11 and 12 individuals, respectively—rather than indicating a true epidemiological pattern.

Understanding the distribution of incidental findings by age can inform clinicians about the likelihood of detecting clinically significant findings in different patient populations, ultimately guiding more tailored diagnostic strategies.

Statistical analysis was performed using JASP (Version 0.19.3, University of Amsterdam) and GraphPad QuickCalcs online service was used for statistical analyses (GraphPad Software, San Diego, CA, USA; available at https://www.graphpad.com/). Categorical variables were summarized as counts and percentages, and proportions were reported with 95% confidence intervals. Group differences across age categories were tested using the chi-square test. A two-sided *p* value < 0.05 was considered statistically significant.

## 3. Results

Altogether, 665 incidental findings were identified in 410 of the 607 patients, corresponding to 67.5% (95% CI 63.7–71.1) of the total cohort. Of the 665 findings, 12 (1.8%; 95% CI 0.9–3.1) were classified as clinically significant (Table 2). When stratified by age group, the proportion of clinically significant incidental findings did not differ significantly across categories (χ^2^ = 5.76, df = 7, *p* = 0.57). The proportion of patients with non-significant incidental findings likewise showed no statistically significant variation between age groups (χ^2^ = 3.21, df = 7, *p* = 0.87). On average, 1.6 incidental findings per affected patient were reported.

**Figure 2:**
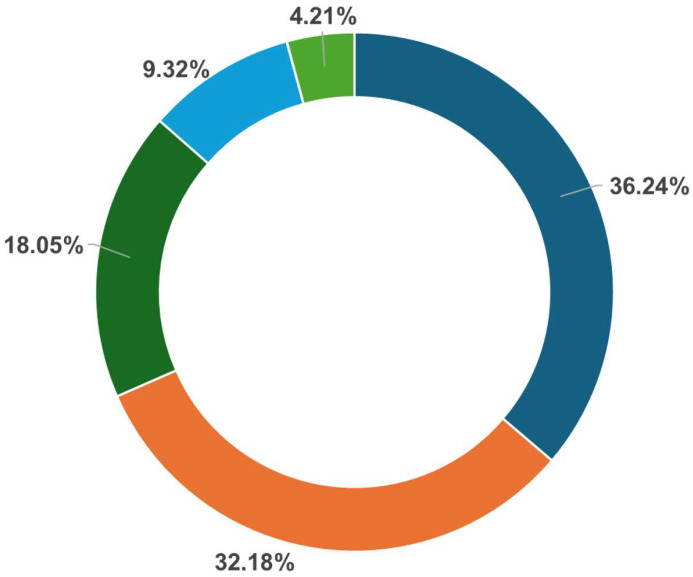
Distribution of Clinical Cases by System Involvement; Neurovascular, Musculoskeletal (MSK), Gastrointestinal (GIT), Urogentinal Findings and Others such as Azites.

Among all findings (Figure 2), urogenital findings marked the most prevalent incidental findings with 241 total findings of which 2 were clinically significant (e.g., hydronephrosis). The gastrointestinal tract was the second most common system in which incidental findings were detected, accounting for 214 cases. Of these, six were clinically significant—including one case of rectal carcinoma and five cases of diverticulitis—classified as Type 1a according to the CDD classification [18]. A total of 120 musculoskeletal incidental findings were described, of which three were clinically significant: one case of sarcoma, one case of avascular necrosis of the femoral head, and one case of high-grade disc protrusion accompanied by severe spinal canal stenosis. Among the 62 neurovascular findings, only one was deemed clinically significant—a case of aortic aneurysm.

**Figure 3:**
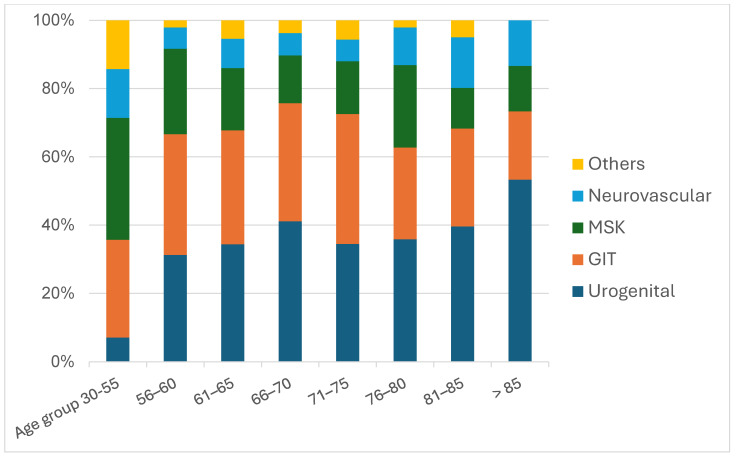
Incidental Findings sorted by System Involvement, Neurovascular, Musculoskeletal (MSK), Gastrointestinal (GIT), Urogentinal Findings and Others such as Azites, and divided across Age Groups.

Apart from urogenital findings—116 of which were diagnosed in patients over the age of 70, demonstrating an age-related increase—the overall distribution of incidental findings remained largely proportional across age groups (Figure 3).

**Figure 4:**
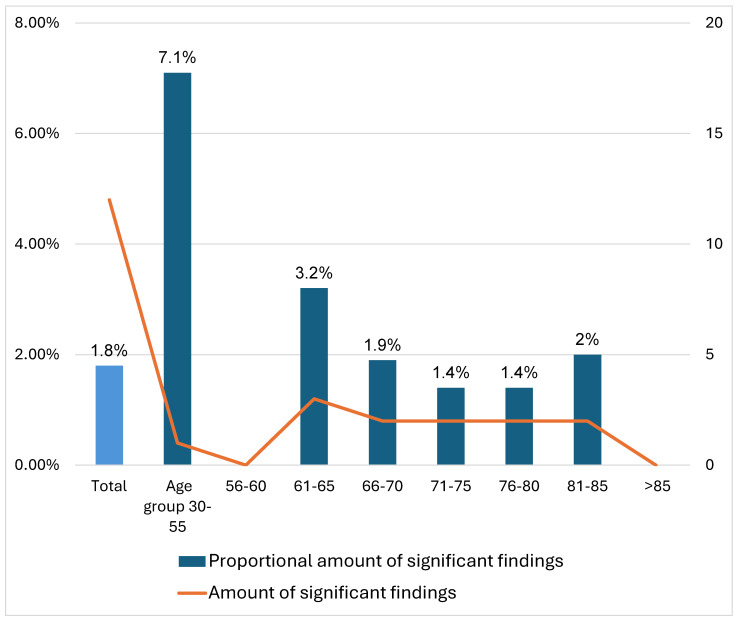
Proportional and Absolute Distribution of Significant Findings by Age Group.

Furthermore, the percentage of clinically significant incidental findings did not increase with age, remaining mostly between 1.4% and 3.2% (Figure 4). Notable outliers were observed in the age groups 30–55 (7.1%), 56–60 (0%) and over 85 (0%), which can be partially attributed to the small sample sizes—only 11 patients under the age of 56 and 12 patients over 85. Interestingly, no significant findings were detected in the 56–60 age group despite a sample size of 46 patients. The presence of such statistical outliers highlights a broader methodological challenge in retrospective imaging studies: the potential for misleading inferences when working with underrepresented subpopulations.

These findings underscore the importance of designing future studies with more balanced age distributions and larger, well-stratified cohorts to improve the reliability of age-specific conclusions.

**Figure 5:**
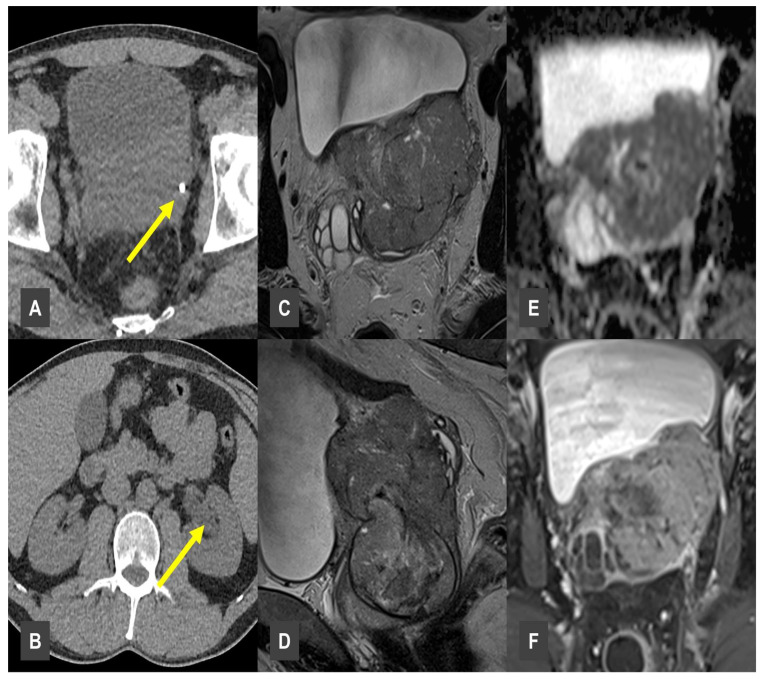
Prostate infiltrating Sarcoma. (**A**,**B**) CT showing urolithiasis with obstructive changes (yellow arrows). (**C**,**D**) MRI demonstrating a large T2w hypointense pelvic mass. (**E**) Diffusion-weighted MRI showing diffusion restriction within the pelvic lesion, suggestive of high cellularity. (**F**) Lesion expressed vivid contrast enhancement.

A particularly case in our study involved a 43-year-old patient who was referred to our clinic due to unexplained flank pain. Initial sonography revealed an unclear intrapelvic mass. A subsequent CT scan confirmed the presence of the intrapelvic mass, as well as a radiopaque structure in the left perivesical region, initially interpreted as a urinary calculus (Figure 5A). Additionally, ipsilateral hydronephrosis was observed (Figure 5B). The lesion was initially diagnosed as prostatitis, based on clinical findings and patient age; however, further workup was recommended to exclude prostate cancer. For further diagnostic evaluation, MRI was performed, which supported the suspicion of a malignant lesion. The MRI revealed a T2-weighted hyperintense, infiltrative pelvic mass, involving the left ureteral orifice (Figure 5C,D). The lesion demonstrated restricted diffusion and marked contrast enhancement (Figure 5E,F). Malignancy was confirmed histologically via biopsy, diagnosing an infiltrating sarcoma.

## 4. Discussion and Conclusions

Our study revealed a high prevalence of incidental findings, observed in 67.5% of patients, highlighting the diagnostic breadth and sensitivity of cross-sectional imaging in routine clinical practice. This finding aligns with previous studies, which have reported incidental finding rates ranging from 40% to 53%. For comparison, Cutaia et al. [12] analyzed 647 patients and identified 461 incidental findings in 341 cases, of which only 12 (1.8%) were deemed definitively clinically significant, including 7 cases of bladder carcinoma. Similarly, Scherrer et al. [11] analyzed imaging data from 580 patients undergoing prostate MRI and detected 349 incidental findings, with 23 (6.6%) considered clinically significant. Notably, the patients in both studies had elevated PSA levels and/or abnormal digital rectal examination (DRE), and in the case of Scherrer et al., the imaging protocol included the upper abdomen, allowing for detection of a broader range of incidental findings. In contrast, the rate of clinically significant findings in our study (1.8%) is notably lower than that reported by Scherrer et al., and we also observed fewer extraprostatic malignant lesions compared to the findings of Cutaia et al. For instance, no cases of bladder carcinoma were identified in our cohort. This may be attributed to the comprehensive pre-imaging clinical evaluations conducted in 402 patients, including prior urine analysis and ultrasound examinations, which likely reduced the likelihood of detecting previously unknown, clinically significant conditions during MRI [19]. In addition, the spectrum of incidental findings in our study shows substantial overlap with those reported in other pelvic MRI examinations in male patients. Tang et al. recently described incidental findings on staging rectal MRI, underscoring that unexpected pelvic abnormalities represent a recurring phenomenon across different MRI indications [20]. Recent literature has also broadened the perspective on unexpected findings in prostate MRI. A comprehensive review by Porões and colleagues highlighted the range of additional findings that can be encountered beyond targeted lesion assessment [21]. Moreover, discordant MRI–biopsy constellations, as analyzed by Stanzione et al., illustrate how unexpected or incidental abnormalities may complicate clinical interpretation and downstream management [22]. Together with our data, these findings emphasize that incidental and unexpected abnormalities represent a recurring and clinically significant aspect of prostate MRI interpretation.

Although some findings such as inguinal hernia, bladder diverticulum, or trabeculation could theoretically be detected by clinical examination or ultrasound, many remain asymptomatic or are overlooked in routine workup. MpMRI may therefore still reveal such conditions unexpectedly, even in patients who had undergone preliminary evaluations.

Beyond extra-prostatic incidental findings in mpMRI, the concept of incidental disease has also been explored in other clinical settings. A recent retrospective study assessed the prevalence and predictors of incidental prostate cancer in patients undergoing surgery for benign prostatic obstruction in the MRI era, demonstrating that unexpected diagnoses can emerge outside the context of prostate mpMRI [23]. Furthermore, radiomics-based approaches have been evaluated for prostate cancer staging, such as the comparison of multiple radiomics models with clinical nomograms for predicting lymph node involvement [24]. While these studies focus on tumor-related outcomes rather than extra-prostatic findings, they illustrate the broader spectrum of incidental diagnoses and AI applications in prostate cancer imaging.

Importantly, our data showed that the overall distribution of incidental findings remained largely uniform across age groups, except for the age-related rise in urogenital findings.

Moreover, aside from the aforementioned outliers, the percentage of clinically significant incidental findings did not increase with age. This statistical analysis confirmed that the distribution of both clinically significant and non-significant incidental findings did not differ significantly between age categories. This supports the notion that clinically meaningful incidental pathology is not confined to older populations, underscoring the importance of thorough image assessment across all age groups [25].

In summary, incidental findings on prostate mpMRI are frequent, but only a minority carry clinical significance. This distinction is important: most findings should not prompt unnecessary diagnostic cascades, but radiologists should remain alert to the subset of incidental findings that may represent serious conditions. Recognizing this balance will help integrate incidental findings into prostate mpMRI reporting in a clinically meaningful way.

Unfortunately, documentation of further diagnostic workup was limited, primarily due to the ambulatory nature of the patient population and the fact that many patients received care externally before and after undergoing fusion biopsy at our center. In addition, the retrospective single-center design and protocol heterogeneity between in-house 1.5 T examinations and heterogenous external mpMRI studies may restrict the generalizability of our findings, and the exclusive use of 1.5 T for in-house imaging could have influenced the detection of smaller incidental findings compared to 3 T systems. Inter-reader agreement was not formally assessed, as all discrepancies were resolved in consensus.

Finally, future research should aim to establish standardized reporting checklists for incidental findings in prostate mpMRI. Such frameworks could improve reporting consistency, facilitate comparability across studies, and reduce unnecessary downstream diagnostics.

## Figures and Tables

**Table 1:** Population Distribution by Age Group.

Age Groups	
**33–55**	n = 11
**56–60**	n = 46
**61–65**	n = 84
**66–70**	n = 103
**71–55**	n = 143
**76–80**	n = 134
**81–85**	n = 74
**>85**	n = 12

**Table 2:** List of clinically significant incidental findings as well as incidental findings with minor clinical significance; n = 665 findings.

Clinically Significant Incidental Findings		Incidental Findings with Minor Clinical Significance	
**Sarcoma**	n = 1	Diverticulosis	n = 207
**Colorectal Carcinoma**	n = 1	Bladder trabeculation	n = 106
**Diverticulitis**	n = 5	Inguinal hernia	n = 79
**Hydronephrosis Grad III**	n = 1	Hydrocele	n = 51
**Priapism**	n = 1	Bladder diverticulum	n = 39
**Aortic Aneurysm with 6 cm diameter**	n = 1	Ascites	n = 27
**Osteonecrosis of the femoral head**	n = 1	Utricular cyst	n = 20
**High grade spine canal stenosis**	n = 1	Other	n = 136

## Data Availability

The datasets generated and analyzed during the current study are not publicly available due to patient privacy regulations and institutional policies. Anonymized data supporting the findings of this study can be obtained from the corresponding author upon reasonable request and with approval of the local ethics committee.

## Abbreviations

The following abbreviations are used in this manuscript:

MRI	Magnetic Resonance Imaging
IF	Incidental Findings
mpMRI	Multiparametric Magnetic Resonance Imaging
